# Supplementary material for: Plurality in multi-disciplinary research: multiple institutional affiliations are associated with increased citations
Source: PeerJ. 2018 Sep 24;6:e5664. doi: 10.7717/peerj.5664 (PMC6160819; doi:10.7717/peerj.5664)
Supplement: Supplemental Information 3 [file peerj-06-5664-s003.docx]

**Supplementary Table:** Linear regression results for modelling the effects of author number and maximum affiliation on citations. Author number reflects the number of authors categorised by quartile.

| **Covariate** | **β Coefficient** | **S.E.** | **95% C.I.** | **t** | **p-value** |
| --- | --- | --- | --- | --- | --- |
| (Intercept) | 1635.83 | 200.60 | 1242.65 – 2029.01 | 8.15 | <0.001 |
| Max. Affiliation = 2 | 0.39 | 0.75 | -1.08 – 1.87 | 0.52 | 0.60 |
| 3 | 1.50 | 1.17 | -0.78 - 3.79 | 1.29 | 0.20 |
| 4 | 3.43 | 2.33 | -1.14 - 7.99 | 1.47 | 0.14 |
| 5 | 5.63 | 4.76 | -3.70 - 14.97 | 1.18 | 0.24 |
| 6 | 7.70 | 11.63 | -15.09 - 30.50 | 0.66 | 0.51 |
| Author Number = 2 | 1.22 | 0.61 | 0.02 - 2.41 | 2.00 | 0.05 |
| 3 | 2.30 | 0.60 | 1.12 - 3.47 | 3.84 | <0.001 |
| 4 | 6.47 | 0.75 | 5.00 - 7.94 | 8.63 | <0.001 |
| Year | -0.81 | 0.10 | -1.01 - -0.62 | -8.13 | <0.001 |
| Journal = Science | 17.78 | 0.41 | 16.98 - 18.59 | 43.35 | <0.001 |
| Journal = Nature | 23.45 | 0.41 | 22.65 - 24.24 | 57.80 | <0.001 |
| Journal = PLOS | -0.40 | 0.78 | -1.93 - 1.13 | -0.51 | 0.61 |
| Max.Affil.=2 : Auth.Num.=2 | 0.09 | 1.00 | -1.88 - 2.06 | 0.09 | 0.93 |
| Max.Affil.=3 : Auth.Num.=2 | -0.11 | 1.49 | -3.04 - 2.81 | -0.07 | 0.94 |
| Max.Affil.=4 : Auth.Num.=2 | -1.92 | 2.86 | -7.53 - 3.69 | -0.67 | 0.50 |
| Max.Affil.=5 : Auth.Num.=2 | -0.81 | 5.63 | -11.85 - 10.23 | -0.14 | 0.89 |
| Max.Affil.=6 : Auth.Num.=2 | 0.38 | 13.98 | -27.02 - 27.78 | 0.03 | 0.98 |
| Max.Affil.=2 : Auth.Num.=3 | 1.19 | 0.94 | -0.66 - 3.04 | 1.26 | 0.21 |
| Max.Affil.=3 : Auth.Num.=3 | 0.43 | 1.38 | -2.28 - 3.13 | 0.31 | 0.76 |
| Max.Affil.=4 : Auth.Num.=3 | -1.52 | 2.63 | -6.67 - 3.62 | -0.58 | 0.56 |
| Max.Affil.=5 : Auth.Num.=3 | -0.77 | 5.23 | -11.03 - 9.49 | -0.15 | 0.88 |
| Max.Affil.=6 : Auth.Num.=3 | -3.74 | 12.28 | -27.81 - 20.33 | -0.30 | 0.76 |
| Max.Affil.=2 : Auth.Num.=4 | 1.87 | 1.06 | -0.20 - 3.94 | 1.77 | 0.08 |
| Max.Affil.=3 : Auth.Num.=4 | 4.30 | 1.43 | 1.49 - 7.10 | 3.01 | 0.002 |
| Max.Affil.=4 : Auth.Num.=4 | 5.93 | 2.57 | 0.90 - 10.96 | 2.31 | 0.02 |
| Max.Affil.=5 : Auth.Num.=4 | 11.91 | 5.02 | 2.06 - 21.75 | 2.37 | 0.02 |
| Max.Affil.=6 : Auth.Num.=4 | -0.90 | 12.00 | -24.42 - 22.63 | -0.07 | 0.94 |
